# Life Goal Content and Health Outcomes: Associations with Mental Health and Health Behaviours Among Turkish University Students

**DOI:** 10.3390/bs16091703

**Published:** 2026-09-21

**Authors:** Gökhan Çakır

**Affiliations:** Faculty of Sport Sciences, Recep Tayyip Erdoğan University, Rize 53100, Türkiye; gokhan.cakir@erdogan.edu.tr

**Keywords:** life goals, goal content, psychological well-being, psychological distress, cognitive flexibility, physical activity, dietary behaviour, university students

## Abstract

Life goals are described as a general psychological resource for health, but whether goals of different content relate equally to mental health and to health behaviour is unknown. Participants were 408 students at a Turkish state university (71.1% women; mean age 21.76 years, SD = 4.20) who completed the Life Goals Scale, distinguishing career, relationship, and body-related goals, together with measures of psychological well-being, psychological distress, cognitive flexibility, physical activity, and nutritional risk. Regression models adjusted for gender, age, grade level, body mass index, and faculty used heteroscedasticity-consistent standard errors, and the two domains were compared using case bootstrapping. Goal setting was associated with all five indicators (β = 0.110 to 0.384), and the association was stronger for the mental health block than the behavioural block (Δβ = 0.129, 95% CI [0.049, 0.205]). However, this difference was carried by cognitive flexibility. Specifically, with that indicator excluded, the contrast fell to 0.077 and was no longer reliable. The two behavioural coefficients did not differ (Δβ = 0.086), and the association with nutritional risk was small and weakly supported. With the three subscales entered simultaneously, physical activity was related to body-related goals alone (β = 0.227, *p* < 0.001) and not to career (β = 0.032) or relationship goals (β = 0.012). Among the three goal contents assessed, what a goal is about, rather than goal setting as such, appears to distinguish which health behaviour it accompanies.

## 1. Introduction

Life goals have occupied psychologists and health researchers for several decades, largely because goal pursuit tends to appear alongside resilience, subjective happiness, and well-being (Baumeister & Vohs, 2002; Ryff, 1989; Steger, 2009). The term is used here in a narrower sense than in parts of that literature: life goals are lasting, future-directed aims that a person sets and then works toward. Purpose and meaning in life are closely related constructs. However, they describe an orientation, whereas goals describe targets. The instrument used here measures goal setting rather than existential meaning. Goals in this sense are usually treated as motivational structures that give a life direction and stability over time (Fang et al., 2024; Schmuck & Sheldon, 2001), and Klinger (1998) argued that they buffer against psychopathological processes, a claim consistent with treating goals as a key psychological resource for well-being (Bühler et al., 2019).

Setting personally meaningful life goals has also been linked to better quality of life and to healthier, more proactive behaviour (Shi, 2025), and Eryılmaz (2012) related goals both to recovery from mental disorder and to psychological and physical health. Goals in this sense are not only a theoretical construct but something a student can be helped, quite practically, to formulate.

University is the period during which many people first have to decide what they are aiming at, which is why this stage shapes identity, decision making, and health at the same time (Hill et al., 2016). Turkish students have faced a difficult decade in this respect. Economic instability, social uncertainty, and recent earthquakes have made the future harder to plan for, and a student who cannot picture a future may struggle to commit to one.

Two features of the existing literature motivate the present design. First, studies in this area usually examine one health outcome at a time, which makes it difficult to establish whether goals matter equally across domains; examining five indicators within a single sample permits a comparison that separate studies cannot deliver. Second, most studies treat life goals as a single global construct, even though work on goal content has long shown that goals differ systematically in what they are about and that these differences carry distinct correlates (Grouzet et al., 2005; Kasser & Ryan, 1996). If goal content matters, a global goal score may obscure associations that are specific to one kind of goal. The design is cross-sectional, so every hypothesis below is stated as an association, and each is motivated by the mechanism the relevant literature proposes rather than by the fact that a significant coefficient has been reported before.

### 1.1. Life Goals and Mental Health

Mental health cannot be reduced to the absence of disease; it also encompasses positive mood and individual development, which is why research in this area increasingly examines mental illness and positive mental health as distinct but related dimensions (Kavussanu et al., 2024). Qiu et al. (2025) extend this view, arguing that mental health is shaped as much by emotional and cognitive functioning as by the presence or absence of psychological disorders, and the present study accordingly treats mental health as a multidimensional construct represented by psychological well-being, psychological distress, and cognitive flexibility. Cognitive flexibility is placed in this set deliberately, and the choice is open to argument since it can equally be read as a resource that supports mental health rather than as an aspect of it. Because this is a theoretical decision rather than a settled matter, the domain comparison is repeated below with cognitive flexibility removed (Section 3.7).

Psychological well-being is defined as the development of an individual’s true potential from a eudaimonic perspective (Ryff, 1995; Ryff & Singer, 2008). It is an important factor in students’ adaptation to university (Morales-Rodríguez et al., 2020), a transition that coincides with a period of heightened vulnerability to declines in well-being and that many universities now address directly (Brooker & Woodyatt, 2019). Life goals are regarded as a positive psychological resource in this context because they supply direction and sustained motivation (Huo et al., 2020; Seligman, 2011; Zhu et al., 2022). Ryff’s (1989) model puts the claim more strongly, treating purpose in life as a constituent of psychological well-being rather than as one of its correlates. If that is right, students who report clearer goals should also report higher well-being, and the first hypothesis follows.

**H1.**  *Higher life goal scores will be associated with higher psychological well-being.*

Psychological distress, a second indicator of mental health, is a state of emotional suffering characterised by symptoms of depression and anxiety (Drapeau et al., 2012) and is common among university students worldwide (Sharp & Theiler, 2018). The mechanism usually proposed is one of stability: a student who knows what they are working toward retains a reference point when circumstances deteriorate, and that reference point is thought to support coping (Klinger, 1998; Zhang et al., 2018). The evidence fits this account. Higher purpose accompanies lower distress (Zhang et al., 2018), and a meta-analysis reports a moderate negative association between meaning in life and distress (He et al., 2023). Mascaro and Rosen (2006) observed the reverse pattern in people who lacked direction. Frankl (1963) reached the same conclusion from the opposite side, treating the absence of goals as itself a source of depression and hopelessness.

**H2.**  *Higher life goal scores will be associated with lower psychological distress.*

Cognitive flexibility, treated here as the cognitive component of mental health, is defined as the capacity to recognise multiple alternatives, generate different solutions and perspectives, and adapt to changing situations (Martin et al., 1998). The reason to expect a link with goals is procedural rather than merely empirical: pursuing a goal obliges a person to monitor progress and to change means when the first route is blocked, which is the operation that measures of flexibility are built to capture (Chevalier & Blaye, 2009; Locke & Latham, 2019). Earlier findings are consistent with this, linking cognitive flexibility to educational attitudes (de-la-Peña et al., 2021), to mental health more broadly (Huang et al., 2021), and to meaning and resilience (Malo et al., 2024; Modzelewska, 2025).

**H3.**  *Higher life goal scores will be associated with greater cognitive flexibility.*

### 1.2. Life Goals and Health Behaviour

Physical health is a critical determinant of quality of life and functional capacity (Sunde et al., 2021), and physical activity and dietary behaviour represent two of its most fundamental behavioural components (Sandri et al., 2025). Lifestyle changes during the university years leave students increasingly vulnerable to the risks associated with inactivity and unhealthy eating (Çol et al., 2024), while goal-setting theory holds that clearly defined goals organise behaviour and enhance motivation (Bird et al., 2024; Locke & Latham, 2019).

Nutritional habits refer to habitual patterns of food consumption, whereas nutritional risk reflects unhealthy, inadequate, or unbalanced dietary behaviour (World Health Organization, 2021). Students are a high-risk group for largely circumstantial reasons: they move away from home, their schedules become irregular, and their food choices come to depend on what is affordable and nearby (Kirkpatrick et al., 2012). Against this, Hu and Lü (2024) argue that goals strengthen health-related values, and Brassai et al. (2015) found that life goals and the search for meaning predicted both healthy eating and regular exercise in adolescents over time. The prediction therefore rests on a specific claim that goals raise the priority of health-relevant values at the point where a food choice is made and not on the observation that the two variables have correlated before.

**H4.**  *Higher life goal scores will be associated with lower nutritional risk.*

Studies of young adults indicate that physical activity interacts with students’ life goals in ways that can improve overall quality of life (Sigvartsen et al., 2016). The benefits of exercise may depend less on the activity itself than on what it means to the person performing it. Codina et al. (2024) point to goals, psychological need satisfaction, and flow as the factors that separate one hour of movement from another, and motives differ, as do the resulting activity levels and quality of life (Mitchell-Miland et al., 2025). The relationship also appears to be reciprocal. Goals shape how much people move (Rhodes et al., 2016), while movement that produces flow feeds back into the goals themselves.

**H5.**  *Higher life goal scores will be associated with higher physical activity.*

### 1.3. Theoretical Framework and the Present Study

The present study draws on three theoretical accounts, each of which specifies a different mechanism linking goal setting to health outcomes. Social cognitive theory treats behaviour as the joint product of environment and cognition (Bandura, 1986), so that a person holding a clear goal thinks over a longer horizon and recovers better from setbacks. Self-determination theory locates the benefit in need satisfaction since goals a person genuinely owns support autonomy, competence, and relatedness (Deci & Ryan, 2013). Broaden-and-build theory supplies the affective component, arguing that positive emotion and goal pursuit reinforce one another over time (Cohn et al., 2009).

The mechanisms differ. However, the prediction they share is that goals should function as a psychological resource visible in health outcomes, and young adulthood is where this is easiest to test since goals are being formed rather than merely maintained. If goals operate as a general resource, their association should be of similar magnitude for mental health and for health behaviour; if it is confined to outcomes a student can plan and act upon directly, the pattern should be uneven. Deciding between these accounts requires the two sets of coefficients to be compared formally rather than by inspection of their significance levels; the procedure is described in Section 2.5. This comparison is stated as a further hypothesis.

**H6.**  *The association between life goals and the mental health indicators will be stronger than the association between life goals and the behavioural indicators.*

### 1.4. Goal Content

The hypotheses stated so far concern goal setting as a global quantity. Work on goal content indicates that goals differ systematically in what they are directed toward and that these differences carry distinct psychological correlates: intrinsic aspirations such as affiliation and personal growth relate to well-being in a way that extrinsic aspirations such as financial success do not (Kasser & Ryan, 1996), goal contents are organised along stable dimensions across fifteen cultures (Grouzet et al., 2005), and the benefits of goal pursuit depend on how far a goal expresses the person’s own interests and values (Sheldon & Elliot, 1999).

The instrument used in the present study distinguishes three goal contents: career or achievement goals, relationship goals, and recreational or body-related goals (Eryılmaz, 2012). This permits a more specific prediction than a global score allows. If goals influence behaviour partly because they direct attention and effort toward a particular class of activity, the association with physical activity should be carried by goals that concern the body and recreation rather than by goals that concern careers or relationships. To our knowledge, this prediction has not been tested with the present instrument. These three contents do not exhaust what students aim at. Specifically, academic attainment, financial security, personal growth, contribution to others, spirituality, and family life are all absent from it, so the prediction below, and the findings that follow from it, concern these three contents rather than goal content in general.

**H7.**  *The association between life goals and physical activity will be specific to body-related goals rather than to career or relationship goals.*

**Figure 1:**
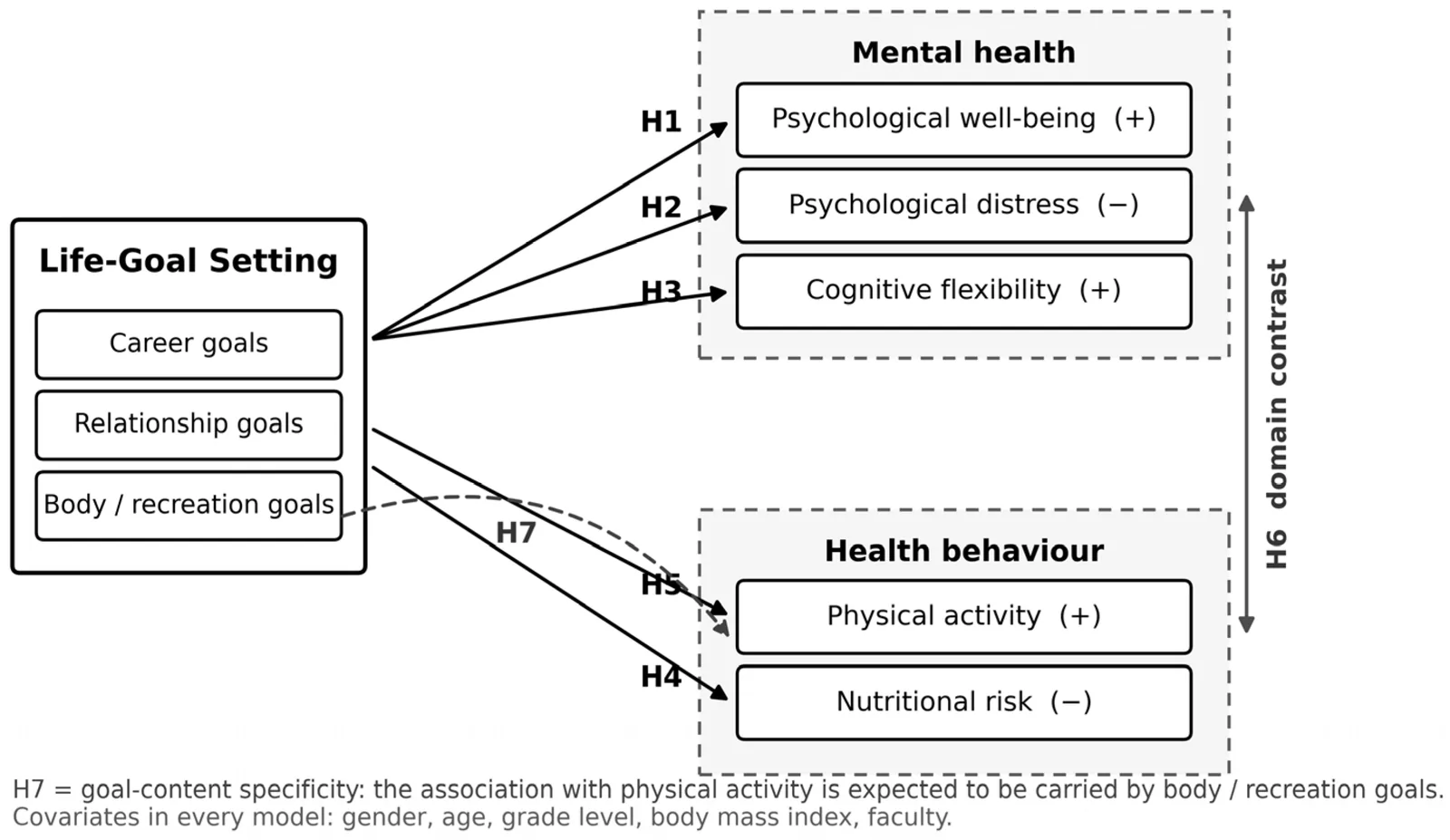
Conceptual model. Signs in parentheses indicate the hypothesised direction of association with life goal setting. H6 denotes the contrast between the two outcome domains; H7 denotes the goal content prediction.

Figure 1 summarises the hypothesised model.

## 2. Materials and Methods

### 2.1. Design

A cross-sectional survey design was used to collect data from university students in Türkiye (İslamoğlu & Alnıaçık, 2019). The design is correlational and predictive. No variable was manipulated and no temporal order was established, so what follows describes associations rather than causes. Life goal setting entered the analyses as a continuous score, with no grouping applied, because dichotomising a continuous predictor discards variance and costs statistical power (MacCallum et al., 2002). Every model carried gender, age, grade level, body mass index, and faculty as covariates; thus, the coefficients reported below express what remains attributable to life goals once these characteristics have been accounted for.

The independent variable is life goal setting, which is examined both as a total score and through its three subscales. The dependent variables fall into two domains: mental health, represented by psychological well-being, psychological distress, and cognitive flexibility as well as health behaviour, represented by physical activity and nutritional risk.

### 2.2. Procedure, Ethics, and Recruitment

Data were collected from students enrolled at Recep Tayyip Erdoğan University during the 2024–2025 academic year. In addition to demographic information, the online survey included scales assessing life goal setting, physical activity, nutritional habits, psychological well-being, psychological distress, and cognitive flexibility. Data collection used a Google Form circulated on social media. Sign-in with a Google account was required, and one response per account was permitted, which ruled out duplicate entries. The form would not submit if any item was left blank, so no submitted response contained missing data.

Of the 432 responses received, 24 were removed because the height or weight values they reported were biologically implausible. The remaining 408 responses were analysed, and no imputation was required. Students read a short statement of the study’s purpose before reaching the scale items and gave informed consent; participation was voluntary throughout. The Social and Human Sciences Ethics Committee of Recep Tayyip Erdoğan University approved the study (24 April 2025, Protocol No. 2025/225).

### 2.3. Participants

The target population was university students in Türkiye, but recruitment was confined to a single province, Rize. Recep Tayyip Erdoğan University had 17,165 registered students when data collection began; for a population of this size, the sample size table of L. Cohen et al. (2018) gives a minimum of 377 at a 95% confidence level with a 5% margin of error. With 408 participants, the study clears that minimum, although the single-province sampling frame remains a constraint and is treated as such in the Limitations section.

No a priori power analysis was carried out before data collection, so a sensitivity analysis was conducted using the achieved sample size and the procedure described by Faul et al. (2007). A sensitivity analysis was preferred over an observed power calculation since the latter is a deterministic function of the *p* value and adds nothing to it (Hoenig & Heisey, 2001). For the test of a single predictor added to a model already containing ten predictors (*N* = 408, df1 = 1, df2 = 397, α = 0.05), the design reaches 80% power for effects as small as f^2^ = 0.019, which corresponds to a standardised coefficient of approximately 0.14. Four of the five effects reported below clear this threshold comfortably. Nutritional risk is the exception, with f^2^ = 0.013 and power of approximately 0.64, and this outcome is interpreted accordingly.

Participants were recruited through convenience sampling. Their characteristics are summarised in Section 3.1.

### 2.4. Measures

#### 2.4.1. Life Goal Setting

The Life Goals Scale, developed within a positive psychotherapy framework by Eryılmaz (2012), was used to determine the life goals of university students. The scale consists of three dimensions, including career or achievement goals, relationship goals, and recreational or body-related goals, and also yields a total score, with higher scores indicating stronger goals in the relevant dimension. The nine-item instrument uses a four-point Likert format ranging from strongly disagree (1) to strongly agree (4). In the original study, the internal consistency coefficient for the whole scale was 0.80 (Eryılmaz, 2012); in the present sample, it was 0.71. Because the goal content hypothesis (H7) concerns the three dimensions separately, the subscales were analysed alongside the total score.

#### 2.4.2. Physical Activity

The Physical Activity Rating Scale (PARS-3), developed by Liang (1994), was used to assess physical activity. The instrument consists of three items covering the intensity, duration, and frequency of activity, and the score is obtained as the product of the three (intensity × duration × frequency), yielding a range from 0 to 100 with higher scores indicating greater activity. No Turkish adaptation of the scale was available. Thus, the original English items were translated into Turkish by the author with expert consultation, and a pretest was administered to check comprehensibility. Internal consistency in the present sample was α = 0.67, with standardised factor loadings of 0.56, 0.74 and 0.62, composite reliability of 0.68, and average variance extracted of 0.41.

Several features of these coefficients require comment before they are interpreted. Because the instrument consists of three items loading on a single factor, the measurement model is saturated (df = 0), and global fit indices cannot be calculated. The average variance extracted falls below the conventional 0.50 threshold, although Fornell and Larcker (1981, p. 46) note that a value below 0.50 may be regarded as acceptable when composite reliability exceeds 0.60, which is the case here. More importantly, the translation carried out for this study did not include back-translation, an independent bilingual panel, or a test of language equivalence, all of which Beaton et al. (2000) treat as standard when a self-report measure crosses cultures. What is reported here is therefore an ad hoc Turkish rendering of a three-item activity index, not a validated Turkish version of the PARS-3, and no claim of validation is made. The implications for interpretation are addressed in the Limitations section.

#### 2.4.3. Nutritional Risk

The six-item Nutritional Habits Index developed by Demirezen and Coşansu (2005) was used to evaluate dietary behaviour. Five items address unhealthy eating behaviours and are scored on a five-point scale from always (4) to never (0); the sixth item concerns the consumption of fruit, vegetables, bulgur, and legumes and is therefore worded in the healthy direction and reverse scored. Under this scoring, a higher total indicates a greater accumulation of risky eating behaviour, which is why the variable is labelled nutritional risk throughout and why the note for Table 1 states that higher scores indicate less healthy dietary behaviour.

Two features of this instrument shape how it is treated here. First, a scoring error was identified and corrected during the preparation of this article. Specifically, item 6 had been administered on the same response format as the five risk items and had not been reversed when the total was computed. The evidence for the error was direct, in that the item correlated negatively with the remaining five in the uncorrected file (corrected item–total r = −0.17). Item 6 was reversed as the scoring instructions require, and every analysis reported below uses the corrected total. The correction raised internal consistency from α = 0.50 to α = 0.61 (ω = 0.62) and the mean inter-item correlation from 0.14 to 0.21. The latter is now within the 0.20 to 0.40 band that Briggs and Cheek (1986) recommend for short scales. The original six-item scale reported 0.80.

Second, and more fundamentally, the six items cover distinct eating behaviours that need not co-occur, so the instrument functions as a formative index rather than a reflective scale. For indices of this kind, internal consistency carries little diagnostic weight (Bollen & Lennox, 1991; Diamantopoulos & Winklhofer, 2001), and alpha is reported here chiefly for comparability with earlier research. Because a formative index can conceal offsetting item-level associations, the six items were also analysed separately as outcomes (Section 3.7).

#### 2.4.4. Psychological Well-Being and Psychological Distress

The Psychological Wellbeing and Distress Screener, developed by Renshaw and Bolognino (2017) and adapted to Turkish by Renshaw and Arslan (2019), was used to assess two-dimensional mental health. The instrument consists of two five-item subscales scored on a five-point Likert format. The subscales are evaluated separately, and no total score is computed. Reliability coefficients in the Turkish adaptation were 0.86 for psychological well-being and 0.87 for psychological distress (Renshaw & Arslan, 2019); in the present sample, they were 0.85 and 0.81, respectively.

#### 2.4.5. Cognitive Flexibility

The Cognitive Flexibility Scale developed by Martin and Rubin (1995) and adapted to Turkish by Çelikkaleli (2014) was used. The eleven-item instrument uses a six-point Likert format, contains reverse-worded items and yields a total score, with higher scores indicating greater cognitive flexibility. Its unidimensional structure showed good fit in the adaptation study (Çelikkaleli, 2014). Internal consistency coefficients in the three samples of the adaptation study were 0.74, 0.73, and 0.75; in the present sample the coefficient was 0.82.

#### 2.4.6. Covariates

Gender, age, and grade level were recorded directly. Body mass index was computed from self-reported height and weight. Faculty was recorded as an open-text field and coded into six categories: Education, Theology, Sport Sciences, Law, Economics and Arts & Sciences, and Other. The set of covariates was fixed before any model was estimated. Each variable was included because it is plausibly related both to life goal setting and to the health outcomes, which is the configuration that produces confounding. A variable was not entered merely because it correlated with an outcome since adjustment on that basis can cost precision without removing bias.

Gender, age, and grade level mark position in the student life course. They bear on what students aim at and on how they eat, sleep, move, and appraise their circumstances. All three are determined before the outcomes were measured, so neither reverse causation nor collider stratification is a plausible concern for them. Faculty was included based on the same reasoning. Programme of study is settled before university health behaviour is observed and is strongly related to habitual activity in this sample, where students of sport sciences reported a mean activity score three times that of their peers (Section 3.1). A model omitting programme would therefore risk crediting life goals with an association that belongs to enrolment.

Body mass index needs a more careful statement because its position in the model is not the same for every outcome. It plausibly shapes whether a student forms body-related goals at all, which makes it a candidate confounder; in the dietary model it may equally be a consequence of eating behaviour, in which case adjusting for it amounts to over-adjustment. Rather than settle the question by assertion, both specifications are reported. Removing body mass index and faculty from the nutritional risk model moves the coefficient for life goal setting from −0.110 to −0.104 (Section 3.4), so the substantive conclusion does not depend on the decision. None of the covariates are a plausible consequence of life goal setting, and variance inflation factors did not exceed 1.24 in any model (Section 3.3). Thus, neither collider bias nor multicollinearity appears to threaten the estimates.

Two coding decisions should be recorded because individual coefficients depend on them. Gender was entered as a dummy variable with men as the reference category, so a negative coefficient indicates a lower score among women. Faculty was entered as five dummy variables with the residual Other category as the reference, so each faculty coefficient reported below is a comparison with that group.

### 2.5. Data Analysis

Descriptive analyses were run in IBM SPSS Statistics v27 (IBM Corp., Armonk, NY, USA) and JASP v0.19.3 (JASP Team, University of Amsterdam, Amsterdam, The Netherlands); the regression, resampling, and equivalence analyses were run in Python 3.11 with statsmodels. Internal consistency was assessed with Cronbach’s alpha and, because alpha understates reliability when items are not essentially tau equivalent, with McDonald’s omega, taking 0.60 as the lower bound (Dunn et al., 2014; P. Kline, 2000; McDonald, 1999). Univariate normality was judged from skewness and kurtosis (|Sk| < 3, |Ku| < 10; R. B. Kline, 2016), and bivariate relationships were judged from Pearson correlations.

The analysis had three stages. In the first, five hierarchical regressions were estimated, one per outcome, with the covariates at Step 1 and life goal setting at Step 2. Reported for each model are the incremental variance attributable to life goals (ΔR^2^), unstandardised and standardised coefficients with 95% confidence intervals, and Cohen’s f^2^, read as small, medium, and large at 0.02, 0.15, and 0.35, respectively (J. Cohen, 1988). Because five outcomes were tested, *p* values were adjusted with the Benjamini–Hochberg procedure, and both raw and adjusted values are given (Benjamini & Hochberg, 1995).

In the second stage, the two domains were compared. All variables were standardised, and the signs of psychological distress and nutritional risk were reversed so that a higher value indicated better health on every outcome. The five equations were then estimated jointly, in the manner of a seemingly unrelated regression system (Zellner, 1962). Each domain was summarised by the unweighted arithmetic mean of its standardised coefficients, which treats the indicators within a domain as equally informative; weighting them would have required an ordering of their importance that neither the literature nor these data supply. Three contrasts were formed: the difference between the two behavioural coefficients, the mean coefficient in each domain, and the difference between the two domain means. Confidence intervals and *p* values came from 5000 case-resampling bootstrap replications (Efron & Tibshirani, 1993), with the means recomputed inside each replication so that the correlations among outcomes are carried through. Because an unweighted mean depends on which indicators make up a block, the domain contrast was also recomputed with cognitive flexibility excluded (Section 3.7). This procedure provides the formal test of H6 that an informal comparison of significance levels cannot supply (Gelman & Stern, 2006).

In the third stage, the three life goal subscales were entered simultaneously into each of the five models, providing the test of H7. Coefficients were again standardised and adjusted for the same covariates, and the fifteen resulting tests were corrected with the Benjamini–Hochberg procedure.

Assumptions were checked before the models were interpreted, using variance inflation factors, externally studentised residuals, Cook’s distance, and the Breusch–Pagan test (Tabachnick & Fidell, 2013). HC3 standard errors (White, 1980) were applied to every model since they give valid inference whether or not homoscedasticity holds (Hayes & Cai, 2007), and confidence intervals accompany all point estimates (Cumming, 2014).

Four sets of sensitivity analyses were conducted. The physical activity model was re-estimated after a logarithmic transformation as a gamma generalised linear model with a log link and as a quantile regression at the 0.25, 0.50 and 0.75 quantiles (Koenker & Bassett, 1978). The nutritional risk coefficient was examined with two one-sided tests of equivalence (Lakens, 2017; Lovakov & Agadullina, 2021), and Bayes factors were approximated from the Bayesian information criterion so that evidence for the null could be quantified rather than inferred from a non-significant *p* value (Kass & Raftery, 1995; Wagenmakers, 2007). In addition, Harman’s single-factor test (Podsakoff et al., 2003), covering thirty-nine items, was administered.

## 3. Results

### 3.1. Participant Characteristics

The final sample comprised 408 students. The majority were women (71.1%, *n* = 290). First-year students were the largest group (32.1%, *n* = 131), followed by second-year (29.7%, *n* = 121), third-year (24.5%, *n* = 100) and fourth-year students (13.7%, *n* = 56). Mean age was 21.76 years (SD = 4.20), and mean body mass index was 23.48 kg/m^2^ (SD = 3.96). Students were enrolled in the Faculty of Education (46.6%, *n* = 190), Theology (18.9%, *n* = 77), Sport Sciences (13.2%, *n* = 54), Law (11.3%, *n* = 46), Economics and Arts & Sciences (8.6%, *n* = 35), and other programmes (1.5%, *n* = 6).

The relevance of the last variable is apparent from a simple comparison: students of sport sciences reported a mean physical activity score of 42.07, against 13.96 for students in all other faculties, Welch t(60.9) = 7.23, *p* < 0.001. Faculty was therefore retained as a covariate in every model.

### 3.2. Descriptive Statistics, Reliability, and Correlations

Absolute skewness ranged from 0.06 to 1.67, and absolute kurtosis ranged from 0.16 to 2.09. Thus, no univariate normality violation was indicated by the criteria of R. B. Kline (2016). Cronbach’s alpha ranged from 0.61 for nutritional risk to 0.85 for psychological well-being, and every composite met the 0.60 criterion. The coefficients for the Nutritional Habits Index and for the translated PARS-3 nonetheless remain modest and are taken up again in the Limitations section.

Life goal setting was moderately and positively associated with cognitive flexibility (r = 0.40, *p* < 0.001) and weakly but positively with psychological well-being (r = 0.29, *p* < 0.001) and physical activity (r = 0.24, *p* < 0.001). Its association with psychological distress was weak and negative (r = −0.20, *p* < 0.001), and its association with nutritional risk was very weak (r = −0.10, *p* = 0.044) and did not survive correction for multiple comparisons. Among the outcome variables, physical activity correlated weakly with all three mental health indicators, whereas nutritional risk correlated only with psychological distress (r = 0.23, *p* < 0.001).

### 3.3. Preliminary Analyses

Variance inflation factors for the continuous predictors did not exceed 1.24 in any model, with a value of 1.04 for life goal setting itself. The highest value among the five faculty dummy variables was 3.58, which is well below the conventional threshold of 5, so multicollinearity was not a concern. Between two and five cases per model had externally studentised residuals beyond |3|, and the largest Cook’s distance across all models was 0.078, well below any conventional threshold. Removing these cases left every conclusion unchanged, and all 408 participants were therefore retained. The Breusch–Pagan test was significant for physical activity (*p* < 0.001) but not for the remaining outcomes, and HC3 standard errors were used throughout for consistency.

Harman’s single-factor test was applied to all thirty-nine items of the five instruments. The first unrotated factor accounted for 18.6% of the total variance, far below the 50% threshold conventionally taken to indicate a problem (Podsakoff et al., 2003). Common method variance therefore appears unlikely to account for the associations reported below, although the single-source, single-occasion design means it cannot be excluded entirely.

### 3.4. Life Goal Setting and Each Health Indicator

Table 2 reports the five hierarchical regression models. Life goal setting accounted for additional variance in every outcome once the demographic covariates had been entered.

The strongest association involved cognitive flexibility, for which life goal setting accounted for a further 14.2% of the variance (β = 0.384, f^2^ = 0.175). This represents a medium-to-large effect by J. Cohen’s (1988) benchmarks, so H3 was supported. Psychological well-being came second (ΔR^2^ = 0.071, β = 0.272), supporting H1, and psychological distress moved in the predicted direction (ΔR^2^ = 0.034, β = −0.188), supporting H2. Physical activity was significantly predicted by life goal setting (ΔR^2^ = 0.037, β = 0.196), supporting H5; gender carried the largest covariate weight in this model (β = −0.591, *p* < 0.001, with male students reporting more activity), followed by enrolment in sport sciences (β = 0.729, *p* = 0.006).

Nutritional risk produced the smallest association of the five. In the fully adjusted model, the coefficient reached the conventional threshold (β = −0.110, 95% CI [−0.227, −0.007], *p* = 0.037, q = 0.037). Under a narrower covariate set containing only gender, age, and grade level, the estimate was −0.104 with *p* = 0.057. The two coefficients are almost identical in magnitude, and what separates them is the *p* value rather than any change in effect size. Thus, the two specifications should not be read as producing qualitatively different findings. H4 is therefore supported only in a weak sense. As Section 3.7 shows, the Bayes factor for this outcome is close to one and the equivalence test is also non-significant, so these data do not distinguish sharply between a small association and none at all. The demographic predictors behave differently across the two specifications. Under the narrower set, both grade level (β = 0.153, *p* = 0.004) and age (β = −0.110, *p* = 0.002) were significant. In the fully adjusted model, grade level was not (β = 0.091, *p* = 0.108), while age (β = −0.095, *p* = 0.017) and body mass index (β = 0.127, *p* = 0.014) were.

### 3.5. Comparing the Two Domains

Table 3 reports the domain contrasts. Coefficients were standardised and oriented so that a higher value indicates better health on every outcome, and confidence intervals were obtained from 5000 bootstrap replications.

**Figure 2:**
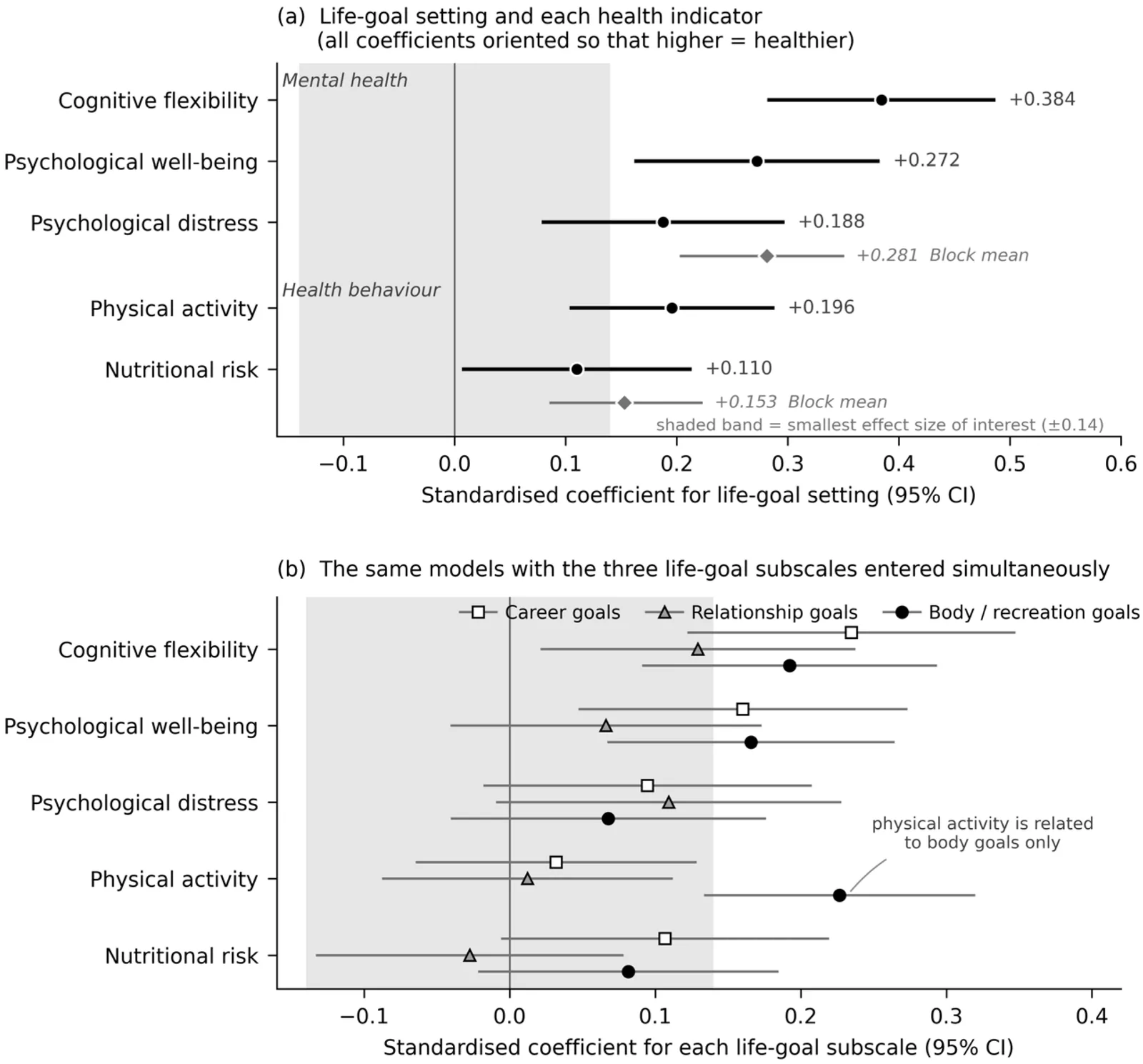
Standardised coefficients with 95% confidence intervals. (**a**) Life goal setting and each health indicator, with all coefficients oriented so that a higher value indicates better health, together with the two block means; the shaded band marks the smallest effect size of interest (±0.14). (**b**) The same models with the three life goal subscales entered simultaneously. All models adjusted for gender, age, grade level, body mass index, and faculty.

The mean standardised coefficient was 0.281 in the mental health domain and 0.153 in the behavioural domain, and the difference between them was reliable (Δβ = 0.129, 95% CI [0.049, 0.205], *p* < 0.001). H6 was therefore supported in that life goals were associated more strongly with mental health than with health behaviour. How far the contrast depends on the composition of the mental health block is examined in Section 3.7. The two behavioural coefficients, however, did not differ from one another (Δβ = 0.086, 95% CI [−0.047, 0.211], *p* = 0.200). The gradient in these data runs between domains rather than between the two behaviours, and the data do not support a categorical distinction under which goals reach physical activity but not diet. Panel (a) of Figure 2 displays these coefficients.

### 3.6. Goal Content

Table 4 reports the models in which the three life goal subscales were entered simultaneously.

The pattern differs sharply across outcomes. Physical activity was associated with body-related goals alone (β = 0.227, q < 0.001), while the coefficients for career goals (β = 0.032) and relationship goals (β = 0.012) were negligible and far from significance. Thus, H7 was supported. Cognitive flexibility, by contrast, drew on career goals (β = 0.235), body-related goals (β = 0.192), and, more weakly, relationship goals (β = 0.129). Psychological well-being drew on career goals (β = 0.160) and body-related goals (β = 0.166). Neither psychological distress nor nutritional risk was associated with any single subscale once the other two were held constant, which suggests that the total score associations reported for these outcomes in Table 2 rest on the accumulation of small contributions rather than on one goal content in particular. The three subscales were only modestly intercorrelated (r = 0.11 to 0.30), so these differences are unlikely to reflect collinearity. Panel (b) of Figure 2 displays the subscale coefficients.

### 3.7. Sensitivity Analyses

The physical activity score was strongly right-skewed (skewness = 1.67), with 10.3% of respondents at the floor, and the Breusch–Pagan test was significant for this outcome. Thus, the model was re-estimated under four further specifications. Table 5 shows that the association with life goal setting is not an artefact of the linear specification.

The quantile results add a detail that the mean-based models conceal. The coefficient increases monotonically across the distribution, so the association between life goals and activity is larger among students who are already comparatively active than among those who are largely sedentary.

Two one-sided tests of equivalence were applied to the nutritional risk coefficient. Using the smallest effect size of interest implied by the sensitivity analysis (β = ±0.14), the equivalence test was not significant (*p* = 0.286), and it remained non-significant under the stricter bound of ±0.10 (*p* = 0.576). The coefficient is thus neither statistically equivalent to zero nor, under the narrower covariate set, distinguishable from it. The Bayes factors point in the same direction. Values approximated from the Bayesian information criterion strongly favoured the alternative hypothesis for cognitive flexibility (BF_10_ ≈ 9.9 × 10^12^), psychological well-being (≈1.1 × 10^6^), physical activity (≈1.7 × 10^3^), and psychological distress (≈1.1 × 10^2^), whereas the value for nutritional risk was 0.71. This is close to one and indicates that the data discriminate weakly between the two hypotheses for this outcome.

Because the Nutritional Habits Index functions as a formative index, the six items were also analysed separately. Two produced significant associations with life goal setting, and they ran in opposite directions. The first item was negatively associated (β = −0.200, q < 0.001), indicating lower risk among students with stronger goals, whereas the sixth item, which concerns the consumption of fruit, vegetables, bulgur, and legumes and is reverse scored, was positively associated (β = +0.134, q = 0.030), indicating lower consumption among the same students. The remaining four items showed no significant association. The weakness of the total score association is therefore partly a matter of offsetting item-level effects, which is precisely the situation in which a formative index is expected to underperform.

Finally, gender was tested as a moderator of every life goal association. None of the five interaction terms approached significance (all q > 0.43), so there is no evidence in these data that the associations differ in magnitude for female and male students. However, the predominantly female sample limits the power of this test.

A final check concerns the composition of the mental health block. Because each block is summarised by an unweighted mean, the size of the domain contrast depends on which indicators the block contains, and cognitive flexibility carried the largest single coefficient in the study. With cognitive flexibility set aside, the mental health block consists of psychological well-being and reversed psychological distress, its mean standardised coefficient is 0.230, and the difference from the behavioural mean of 0.153 falls to 0.077. That reduced contrast is no longer reliable: its bootstrap interval includes zero (95% CI [−0.016, 0.165], *p* = 0.101). The direction of the difference is unchanged, but neither its magnitude nor its statistical reliability survives the removal of the cognitive indicator. The domain contrast reported in Section 3.5 therefore rests substantially on cognitive flexibility, and it is stated below with that qualification.

## 4. Discussion

This study examined how university students’ life goals relate to five mental health and behavioural indicators within a single sample, and it addressed two questions that the existing literature leaves open: whether the association is of comparable size across domains and whether it depends on what the goals are about. Three findings organise the discussion: life goals were associated with all five indicators once demographic characteristics, body mass index, and faculty were accounted for; the association was reliably stronger in the mental health domain than in the behavioural domain; and the association with physical activity was carried entirely by body-related goals.

The largest effect in the study involved cognitive flexibility (β = 0.384, ΔR^2^ = 0.142, f^2^ = 0.175). Students with higher life goal scores were considerably more likely to report generating alternative ways of thinking, which suggests that a sense of direction may matter most for cognitive adaptability and for coping under stress. Chevalier and Blaye (2009) reported a comparable link between goal setting and flexibility, and Kara et al. (2021) found cognitive flexibility, meaning in life, and forgiveness flexibility moving together. On this reading, goals are not merely a matter of having targets; they appear to draw on wider psychological resources related to how flexibly people deploy their cognitive capacities.

Psychological well-being moved with life goals as expected, which matches earlier work relating goals and meaning to well-being (Arslan & Allen, 2022; García-Alandete, 2015). Psychological distress ran in the opposite direction, consistent with the argument of Zhang et al. (2018) that fostering goals supports coping during emotional difficulty. It is worth noting that neither outcome was attributable to a single goal content once the three subscales were entered together, which suggests that what benefits mental health is the presence of goals across domains rather than any particular kind of goal.

The formal comparison supports the view that life goals are more closely associated with mental health than with health behaviour. The mean coefficient was 0.281 in the first domain and 0.153 in the second, and the difference was reliable. The qualification set out in Section 3.7 belongs with that statement. Much of the gap is attributable to cognitive flexibility. With that indicator removed, the difference between the domain means falls to 0.077, and its bootstrap interval includes zero. What these data support, then, is a contrast between the cognitive indicator and the two behavioural ones rather than a clean separation of two domains, and the claim should be read in those narrower terms. This is still the comparison that separate single-outcome studies cannot deliver and stating it as a tested contrast rather than as an inference from differing significance levels avoids the error identified by Gelman and Stern (2006).

The finer distinction fares less well. The coefficients for physical activity and nutritional risk did not differ from one another, so the intuitively appealing account under which goals reach behaviours a student can schedule but not behaviours embedded in daily routine is not supported by these data. The nutritional finding itself requires careful statement. In the fully adjusted model, the association was significant (β = −0.110, *p* = 0.037, q = 0.037). However, the corresponding Bayes factor was 0.71, and the equivalence test was also non-significant. Thus, the evidence is weak in both directions. The most defensible summary is that a small association is present, that its magnitude is near the limit of what this sample can resolve, and that replication in a larger sample is required before it is treated as established.

Three considerations help make sense of the small size of the dietary association. The first is circumstantial. Students’ dietary intake is constrained by campus catering provision, food prices, time pressure, and shared living arrangements (Çol et al., 2024; Kirkpatrick et al., 2012), and these constraints operate largely independently of an individual’s long-term goals. Therefore, intentions translate into behaviour poorly. The second is a matter of measurement since the item-level analysis showed two significant associations running in opposite directions, which is exactly what a formative index built from heterogeneous behaviours would be expected to produce (Bollen & Lennox, 1991). The third concerns the covariates. Body mass index and age, rather than grade level, emerged as the demographic predictors of nutritional risk in the fully adjusted model, which suggests that dietary risk in this sample tracks body composition and age more closely than it tracks time spent at university. The coefficient for life goals itself barely moved when these covariates were added, so what the fuller model changes is the account of which demographic factor tracks dietary risk, not the size of the goal association.

The clearest new finding concerns goal content. Among the three contents this instrument distinguishes, physical activity was associated with body-related goals (β = 0.227) and with neither career nor relationship goals, whose coefficients were effectively zero. A global life goal score therefore misdescribes the relationship it summarises: what accompanies a more active lifestyle is not goal setting in general but goals that concern the body and recreation in particular. The result is consistent with the broader literature on goal content, in which the correlates of a goal depend on what the goal is about rather than on the mere fact of having one (Grouzet et al., 2005; Kasser & Ryan, 1996), and with the argument of Codina et al. (2024) that the psychological benefits of physical activity depend on the goals that motivate it.

The pattern for the mental health outcomes was different in an instructive way. Cognitive flexibility and psychological well-being were associated with career goals as well as with body-related goals, and psychological distress with none of the three individually. Goal content thus appears to matter most where the outcome is a specific behaviour and least where the outcome is a general state, which is what one would expect if the mechanism connecting goals to behaviour runs through the direction of attention and effort toward a particular class of activity (Locke & Latham, 2019). This interpretation is post hoc. It was reached after the coefficients had been inspected. It is not a finding of this study, and it should be treated as a conjecture until an experiment of the kind described below has tested it directly.

The practical consequence is immediate. A goal-setting programme intended to increase physical activity would, on this evidence, need to help students formulate goals concerning the body and recreation specifically; helping them clarify career goals, however valuable for other outcomes, would not be expected to move activity levels.

Although not tied to a specific hypothesis, the interrelations among the outcome variables are worth revisiting given the study’s holistic framing of student health. Physical activity correlated significantly, if weakly, with all three mental health indicators, whereas nutritional risk correlated with psychological distress alone and with neither psychological well-being nor cognitive flexibility. In this sample, the connection between the mental and the physical runs through physical activity rather than through diet. Existing research shows that mental health problems increase the risk of chronic disease while physical illness may contribute to psychological difficulty (Scott et al., 2016), and regular physical activity has been shown to be positively associated with outcomes in both domains (Rebar et al., 2015). Considering mental and physical health processes together, as done here, may therefore support more comprehensive health-related interpretations in young adult populations.

Several limitations qualify these conclusions. The cross-sectional design means that causal relationships between the variables cannot be established, and the reciprocal possibility raised in Section 1.2—that activity feeds back into the goals themselves—cannot be examined with these data. Self-report measurement may introduce social desirability bias. Because all variables were gathered by self-report at a single point in time, residual common method variance cannot be ruled out entirely. However, Harman’s test gave no indication of a problem. The voluntary nature of participation introduces potential self-selection bias since students already invested in health and psychological well-being may have been more likely to enrol.

The sample was drawn from a single state university in one province, which limits generalisability, and cultural specificities may have influenced the observed relationships. Therefore, caution is warranted in extending these findings to other contexts. Instrument reliability is a further limitation. The Nutritional Habits Index (α = 0.61, ω = 0.62) and the Turkish translation of the PARS-3 (α = 0.67; AVE = 0.41) sit at or just above the lower bound of conventional psychometric standards. As stated in Section 2.4.2, the translated activity index is not a validated Turkish instrument. The behavioural results, and the nutritional finding in particular, should be read with this in mind.

Two limitations concern the goal measure itself. The first is one of coverage. The instrument distinguishes career, relationship, and body-related goals, and these three do not represent the whole of what a student aims at. Specifically, academic attainment, financial security, personal growth, contribution to others, spirituality, family life, and social participation are all absent from it. The findings reported here therefore speak to these three contents rather than to goal content in general, and a content the instrument does not cover could stand in a different relation to any of the five outcomes. The second concerns the properties of a goal other than its content. Goal importance, commitment, self-concordance, the balance of autonomous and controlled motivation, perceived attainability, and progress toward the goal were not measured, yet each is known to bear on mental health (Deci & Ryan, 2013; Sheldon & Elliot, 1999). Two students may hold the same body-related goal for quite different reasons, namely, one wanting to be healthy and the other wanting to avoid what others think of their appearance, and the literature gives good reason to expect these two goals to relate differently to well-being and distress. Without these variables the present models describe what students aim at but not why, and part of the unexplained variance in the mental health outcomes may lie there.

Transparency also requires that the scoring correction be recorded here. Item 6 of the Nutritional Habits Index had not been reverse scored in the original data file and was corrected before the analyses presented in this article; all reported values use the corrected scoring. A related caveat concerns statistical power. The sensitivity analysis put power at approximately 0.64 for an effect of the size observed for nutritional risk, and the Bayes factor for that outcome was close to one. Thus, this particular result is best treated as provisional.

Finally, several potentially relevant variables were not measured, among them socioeconomic status, social support, self-esteem, personality traits, and resilience. Their omission raises the risk of omitted variable bias. The sample also remained predominantly female (71.1%). Gender was tested as a moderator, and no interaction was detected. However, the imbalance limits the power of that test.

These findings carry two implications for practice. The first concerns what goal-based work in university counselling services can reasonably be expected to achieve. Programmes that help students articulate and structure their goals may plausibly improve psychological well-being, reduce distress, and support cognitive flexibility since these were the outcomes on which life goals carried the greatest independent weight. Their effect on health behaviour should be expected to be smaller, and, in the case of physical activity, to depend on whether the goals addressed concern the body at all.

The second concerns targeting. In the fully adjusted model, body mass index rather than grade level predicted nutritional risk. This argues against interventions premised on the idea that dietary problems accumulate simply with time spent at university and in favour of approaches that address the material environment: what campus catering offers, what it costs, and whether it is available at the hours students actually eat. Physical activity provision deserves separate mention since it was the only behaviour connected to all three mental health indicators and may therefore return benefits in both domains at once.

For counselling practice, the distinction takes a concrete form. A student who presents with low mood and a sense of drift is likely to benefit from goal work of almost any content since the three mental health indicators were associated with goals across the board rather than with one kind of goal in particular. A student whose presenting difficulty is inactivity is a different case because the association with activity ran through body-related goals alone; work that clarifies career plans, valuable as it may be in other respects, should not be expected to change how much that student moves. Goal-setting protocols used in student services could be adjusted accordingly, with goal content assessed at intake rather than assumed, and with the two kinds of presentation handled differently from the outset.

A third implication is academic and bears on how this literature reports its results. Global life goal scores are convenient, but these data show that such a score can average one strong content-specific association together with two null ones and present the result as a modest general effect. Studies that use composite goal measures without examining content therefore risk understating the associations they are looking for and misplacing them, which matters for anyone who designs an intervention on the strength of them. Reporting subscale coefficients alongside the total score costs little and would make findings across studies easier to compare. The same argument applies to the outcome side. Because the domain contrast reported here depends appreciably on which indicators define the mental health block, studies that summarise a domain by a composite would do well to show how that composite is built and how the result changes when it is built differently.

Several directions for future research follow. The goal-content finding invites the most direct follow-up. If goals shape behaviour through the direction of attention and effort, then a goal-setting intervention that manipulates goal content—body-related in one condition, career-related in another—should move physical activity in the first condition only. Such a design would convert the present correlational result into a causal test, and it would do so at modest cost.

Measurement is the second priority. A properly adapted Turkish PARS-3, built with back-translation, an independent bilingual panel, and formal equivalence testing, would remove one of the clearest constraints on these findings. Dietary behaviour needs comparable attention since a brief index of six heterogeneous behaviours cannot capture what students eat with much precision. Future work would gain from 24-h recalls, food diaries, or other records that do not rest on a single self-report score. Broader sampling across provinces and institution types, together with the covariates omitted here, would strengthen whatever conclusions such data support.

One boundary condition deserves testing in its own right. Goals are treated throughout this literature as beneficial, yet their value may depend on whether they are attainable given a person’s capacities, values, and material circumstances. Where goals and circumstances are badly matched, failure to realise them may produce disappointment, burnout, and falling self-esteem rather than the protective effects described above (Emmons, 2003; Sheldon & Elliot, 1999). The possibility is especially relevant in economically strained settings, where ambitious goals may be held by students with little realistic prospect of reaching them, and whether goal–capacity fit moderates the associations reported here is an open and, in the present context, a pressing question.

## 5. Conclusions

Life goal setting was associated with all five health indicators examined here once gender, age, grade level, body mass index, and faculty were accounted for. In addition, the associations were reliably stronger for the mental health indicators than for the two behavioural indicators. However, that contrast depended on cognitive flexibility and did not survive its removal. The two behavioural coefficients did not differ from one another, so these data give no support to a categorical distinction between activity and diet. The association with nutritional risk was small and its evidential strength weak, and it should be regarded as provisional. The clearest result concerns goal content. Among the three contents assessed, physical activity was associated with body-related goals alone and not with career or relationship goals, which indicates that a global life goal score can obscure the very association it is taken to summarise. These conclusions remain provisional. The design is cross-sectional, and two of the measures are psychometrically modest. In addition, the sample was drawn from a single state university in one province, so how far the pattern holds in other institutions, regions, and student populations is untested. Longitudinal and experimental work will be needed before anything can be said with confidence about direction or causality.

## Figures and Tables

**Table 1:** Descriptive statistics, reliability coefficients, and correlations among the study variables.

Variable	M	SD	α	ω	1	2	3	4	5
1. Life goal setting	24.67	3.54	0.71	0.66	—				
2. Psychological well-being	14.57	3.67	0.85	0.85	0.288 ***	—			
3. Psychological distress	12.26	4.25	0.81	0.79	−0.195 ***	−0.359 ***	—		
4. Cognitive flexibility	48.66	6.98	0.82	0.84	0.396 ***	0.311 ***	−0.197 ***	—	
5. Nutritional risk	13.44	3.77	0.61	0.62	−0.100 *	−0.065	0.230 ***	−0.024	—
6. Physical activity	17.68	21.79	0.67	0.68	0.242 ***	0.253 ***	−0.153 **	0.216 ***	−0.081

Note. Higher nutritional risk scores indicate less healthy dietary behaviour. Nutritional risk is based on the corrected scoring described in Section 2.4.3. α = Cronbach’s alpha; ω = McDonald’s omega. * *p* < 0.05, ** *p* < 0.01, *** *p* < 0.001.

**Table 2:** Hierarchical regressions predicting the five health indicators from life goal setting.

Outcome	R^2^ Step 1	ΔR^2^	B (SE)	95% CI	β	t	*p*	q	f^2^
Cognitive flexibility	0.046	0.142	0.757 (0.103)	[0.555, 0.959]	0.384	7.34	<0.001	<0.001	0.175
Psychological well-being	0.105	0.071	0.282 (0.058)	[0.168, 0.397]	0.272	4.83	<0.001	<0.001	0.087
Physical activity	0.262	0.037	1.204 (0.289)	[0.637, 1.771]	0.196	4.16	<0.001	<0.001	0.053
Psychological distress	0.080	0.034	−0.225 (0.067)	[−0.356, −0.094]	−0.188	−3.37	<0.001	0.001	0.038
Nutritional risk	0.098	0.012	−0.117 (0.056)	[−0.227, −0.007]	−0.110	−2.09	0.037	0.037	0.013

Note. Step 1 comprises gender, age, grade level, body mass index, and faculty; Step 2 adds life goal setting. Coefficients, standard errors, confidence intervals, and t values are based on heteroscedasticity-consistent (HC3) standard errors. q = *p* value adjusted with the Benjamini–Hochberg procedure across the five Step 2 tests.

**Table 3:** Standardised coefficients and domain contrasts from the jointly estimated model.

Parameter	Estimate	95% CI	*p*
Cognitive flexibility	0.384	[0.287, 0.467]	<0.001
Psychological well-being	0.272	[0.163, 0.371]	<0.001
Psychological distress (reversed)	0.188	[0.077, 0.291]	<0.001
Physical activity	0.196	[0.110, 0.281]	<0.001
Nutritional risk (reversed)	0.110	[0.011, 0.214]	0.029
Mental health block, mean	0.281	[0.204, 0.350]	<0.001
Behavioural block, mean	0.153	[0.087, 0.222]	<0.001
Domain contrast (mental−behavioural)	0.129	[0.049, 0.205]	<0.001
Physical activity−nutritional risk	0.086	[−0.047, 0.211]	0.200

Note. All coefficients are oriented so that a higher value indicates better health. All models adjusted for gender, age, grade level, body mass index, and faculty. The mental health mean includes cognitive flexibility; Section 3.7 reports the same contrast with that indicator excluded.

**Table 4:** Standardised coefficients for the three life goal subscales entered simultaneously.

Outcome	Career Goals β (q)	Relationship Goals β (q)	Body/Recreation Goals β (q)
Cognitive flexibility	0.235 (<0.001)	0.129 (0.046)	0.192 (<0.001)
Psychological well-being	0.160 (0.015)	0.066 (0.276)	0.166 (0.003)
Physical activity	0.032 (0.593)	0.012 (0.809)	0.227 (<0.001)
Psychological distress	−0.095 (0.162)	−0.109 (0.130)	−0.068 (0.276)
Nutritional risk	−0.107 (0.130)	0.027 (0.651)	−0.082 (0.178)

Note. All models adjusted for gender, age, grade level, body mass index, and faculty, with HC3 standard errors. q = *p* value adjusted with the Benjamini–Hochberg procedure across the fifteen tests.

**Table 5:** Physical activity: coefficient for life goal setting under alternative model specifications.

Specification	Coefficient	SE	*p*
Ordinary least squares with HC3 (standardised)	0.196	0.047	<0.001
Log-transformed outcome, HC3 (standardised)	0.225	0.043	<0.001
Gamma generalised linear model, log link	0.298	0.051	<0.001
Quantile regression, τ = 0.25 (unstandardised)	0.414	0.135	0.002
Quantile regression, τ = 0.50 (unstandardised)	0.727	0.187	<0.001
Quantile regression, τ = 0.75 (unstandardised)	1.835	0.403	<0.001

Note. All specifications adjusted for gender, age, grade level, body mass index, and faculty.

## Data Availability

The dataset generated and analysed during the current study is available from the corresponding author on reasonable request.

## Abbreviations

The following abbreviations are used in this manuscript:

AVE	Average variance extracted
BF	Bayes factor
CI	Confidence interval
HC3	Heteroscedasticity-consistent covariance estimator 3
PARS-3	Physical Activity Rating Scale-3
